# Liver cirrhosis is a risk-factor for Pneumocystis jirovecii associated mortality

**DOI:** 10.3389/fmed.2024.1474835

**Published:** 2024-10-09

**Authors:** Georg Peschel, Nils Happ, Jan Bornschein, Florian Weis, Stephan Schmid, Martina Mueller, Michael Selgrad

**Affiliations:** ^1^Department of Internal Medicine I, University Hospital of Regensburg, Regensburg, Germany; ^2^Department of Gastroenterology and Oncology, Hospital of Fürstenfeldbruck, Fürstenfeldbruck, Germany; ^3^Translational Immune Discovery Unit, MRC Weatherall Institute of Molecular Medicine, Univeristy of Oxford, John Radcliffe Hospital, Oxford, United Kingdom; ^4^Department of Anaesthesiology-Grosshadern, University Hospital Munich, Munich, Germany; ^5^Department of Anaesthesiology, Hospital of Fürstenfeldbruck, Fürstenfeldbruck, Germany

**Keywords:** liver cirrhosis, Pneumocystis jirovecci infection, mortality, MELD score, cirrhosis associated immune dysfunction

## Abstract

**Background:**

Pneumocystis jirovecci pneumonia (PCP) is a life threating disease in immunodeficient patients. Liver cirrhosis itself can lead to immunodefiency, however little is known if Pneumocystis jirovecci infection affects the outcome of patients with liver cirrhosis.

**Aim:**

We aimed to assess the predictors for Pneumocystis jirovecci-associated mortality in patients with Pneumocystis jirovecci infection treated at intensive care units.

**Methods:**

A total of 151 patients hospitalized between January 2013 and November 2019 with a PCR-confirmed Pneumocystis jirovecci infection were retrospectively included in this study and analysed for clinical predictors for PCJ associated mortality.

**Results:**

The overall mortality in our patient cohort was 60%. Out of 151 patients included in the analysis, 67 (44%) patients suffered from liver cirrhosis. Patients with an advanced liver cirrhosis (Child-Pugh class C) showed the highest mortality rate of 84.7%. The presence of a liver cirrhosis was associated with a significant increased risk of mortality (OR: 4.809) ([95%-CI: 2.32–9.97]; *p* < 0.001). There was a significant correlation of Meld score and mortality (*r* = 0.612, *p* < 0.001).

**Discussion:**

To our knowledge, this study represents the largest evaluation of Pneumocystis jirovecci infection in patients with advanced liver cirrhosis. Cirrhosis associated immune dysfunction (CAID) describes the spectrum of immunological disturbances in patients with cirrhosis, which is linked to a heightened vulnerability to bacterial infections. Our data indicate a heightened susceptibility to fungal infections. Understanding the phenotypic manifestations of CAID could lead to immune-targeted therapies aimed at reducing infection susceptibility and decreasing CAID-associated mortality in cirrhosis patients.

## 1 Introduction

Pneumocystis jirovecci pneumonia (PCP) is an opportunistic fungal infection that can be life-threatening in immunodeficient patients (1). Most often affected are patients with HIV infection (2). However, in recent years, there has been an increase in the incidence of PCP in non-HIV patients (3, 4). Thus, various risk factors other than HIV have been identified including hematologic malignancies, immunosuppressive therapy, solid organ transplantations, and autoimmune diseases (3, 5).

Liver cirrhosis (LC) leads to an impairment of the immune system called cirrhosis-associated immune dysfunction (CAID) (6–9). The term refers to the main syndromic abnormalities of immune function, immunodeficiency and systemic inflammation that are present in LC. The natural course of advanced LC, regardless of its etiology, is often complicated by CAID with this constituting the pathophysiological hallmark of an increased susceptibility to bacterial infections (10).

Up to now, liver cirrhosis is not considered to be a typical risk factor for PCP and little is known about the mortality of PCP in patients with liver cirrhosis.

In the current study, we analyzed predictors for Pneumocystis jirovecii associated mortality in patients with Pneumocystis jirovecci infection treated at intensive care units (ICU) of a German university hospital.

## 2 Patients and methods

### 2.1 Study cohort

A total of 151 patients were retrospectively enrolled in this study. These patients were treated either at a gastroenterological and hepatological (*n* = 103) or cardiological (*n* = 48) ICU of the University Hospital of Regensburg, Germany. The electronic patient documentation system of the University Hospital of Regensburg, Germany, was used to retrieve clinical data of all patients that were diagnosed with microbiologically diagnosed Pneumocystis jirovecci infection as outlined below in the period between January 2013 and November 2019. All patients with a positive microbioligical test for Pneumocystis jirovecci infection treated at one of the two ICU’s were included. There were no exclusion criteria, however three patients were excluded due to a transfer to another hospital and therefore a follow-up was not possible. Two further patients were also excluded due to liver transplantation during their stay at the ICU. The study was approved by the local ethics committee and conducted according to the ethical guidelines of the Declaration of Helsinki as revised in 1989.

### 2.2 Microbiological assessment of Pneumocystis jirovecci

Microbiological identification of Pneumocystis jirovecci was defined as a positive nested real time polymerase chain reaction (PCR) on samples from throat rinse water, tracheal secretion, bronchial secretion or broncho-alveolar lavage (BAL). A sample was considered to contain *Pneumocystis* DNA if the nested PCR was positive on repeated testing (11, 12). The positive microbiological results were quantitatively classified in to six groups (see Table 1).

**Table 1:** Specific Pneumocystis jirovecci therapy in correlation with the pathogen concentration.

		Total (*n*) (%)	No therapy (*n*)	Specific therapy (*n*)	
		151	47	104 (70%)	
Fungal pathogen concentration	<100	20 (13%)	14 (30%)	6 (6%)	*p* = 0.365
	100–1,000	34 (23%)	16 (34%)	18 (17%)	
	1,000–10.000	61 (40%)	14 (30%)	47 (45%)	
	10.000–100.000	18 (12%)	2 (4%)	16 (15%)	
	100.000–1.000.000	9 (6%)	1 (2%)	8 (8%)	
	>1.000.000	9 (6%)	0 (0%)	9 (9%)	

### 2.3 Statistical analysis

All statistical analyses were performed using SPSS22.0 for windows (IBM SPSS Statistics, IBM Corp., United States). The Mann–Whitney U test was used for non-parametrical comparisons. Where appropriate, categorical data were compared by Chi^2^-test and Fisher’s exact test also applying Spearman’s rank correlation coefficient for risk assessment. For all tests a two-sided significance level of *p* < 0.05 was considered as statistically significant.

## 3 Results

### 3.1 General characteristics of the study population

Of the 151 individuals enrolled in this study, 53 (35%) were women and 98 (65%) were men with a median age of 58.0 years (±13.5 years). Sixty-seven (44%) of the patients were diagnosed with LC, 8 (12%) of them in the Child-Pugh class B and 59 (88%) in Child-Pugh class C. The genesis of the LC was mainly ethyltoxic (see Supplementary Figure S1). Invasive ventilation was administered to 141 patients (93%), non-invasive ventilation was used for 4 patients (3%), and 6 patients (4%) were not ventilated. The clinical characteristics of the patient cohort study are summarized in Table 2.

**Table 2:** Patient characteristics and main clinical data.

		Total (*n*) (%)	Survivor	Dead	Statistics
Number of patients		151	61	90	
Sex	Men	98 (65%)	36 (59%)	62 (69%)	*p* = 0.212
	Women	53 (35%)	25 (41%)	28 (31%)	
Microbiological specimens	Throat rinse water	6 (4%)	4 (7%)	2 (2%)	*p* = 0.218
	Tracheal secretion	36 (24%)	14 (23%)	22 (24%)	
	Bronchial secretion	21 (14%)	5 (8%)	16 (18%)	
	Broncho–Alveolar lavage	88 (58%)	38 (62%)	50 (56%)	
Fungal pathogen concentration	<100	20 (13%)	11 (18%)	9 (10%)	*p* = 0.365
	100–1,000	34 (23%)	15 (25%)	19 (21%)	
	1,000–10.000	61 (40%)	23 (38%)	38 (42%)	
	10.000–100.000	18 (12%)	5 (8%)	13 (14%)	
	100.000–1.000.000	9 (6%)	2 (3%)	7 (8%)	
	>1.000.000	9 (6%)	5 (8%)	4 (4%)	
LC	No LC	84 (56%)	47 (77%)	37 (41%)	*p* = 0.001, OR = 4.809 (2.32; 9.97)
	LC	67 (44%)	14 (23%)	53 (59%)	
Child-pugh classification	Child B	8 (12%)	5 (36%)	3 (6%)	*p* = 0.002 OR = 9.259 (1.87; 45.75)
	Child C	59 (88%)	9 (64%)	50 (94%)	
Meld score		32.3 (±7.8)	23.1 (±7.5)	34.8 (±5.8)	*p* < 0.001
Ventilation	None	6 (4%)	5 (8%)	1 (1%)	*p* = 0.30
	Non invasive	4 (3%)	3 (5%)	1 (1%)	
	Invasive	141 (93%)	53 (87%)	88 (98%)	
Immunosupression		77 (51%)	32 (21%)	45 (58.4%)	*p* = 0.767
	Auto-immune disease	40	16 (40%)	24 (60%)	
	Malignancy	23	6 (26%)	17 (74%)	
	Post transplant	6	4 (67%)	2 (33%)	
	HIV infection	9	7 (78%)	2 (22%)	

### 3.2 Predictors for mortality

**Figure 1:**
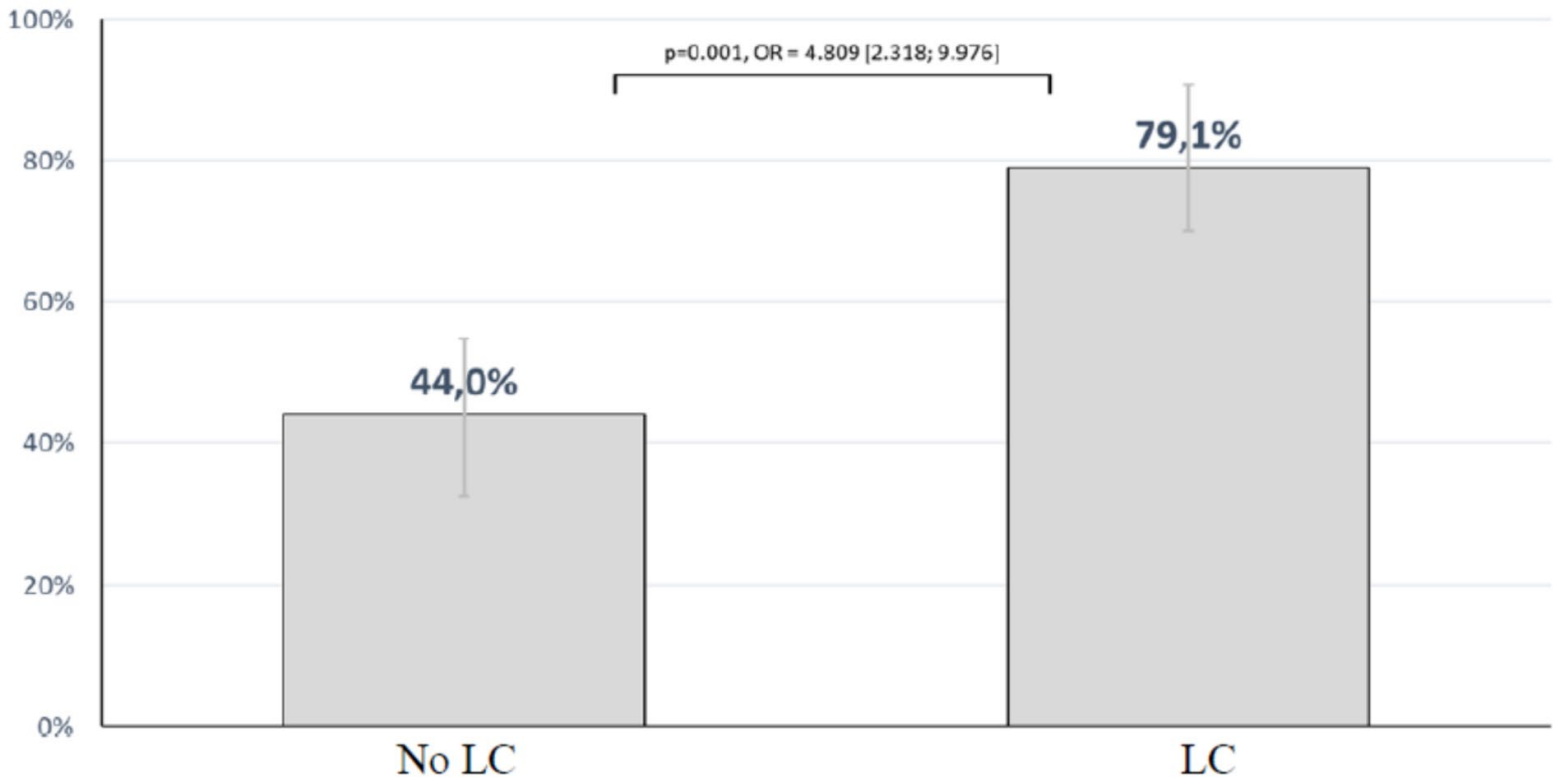
Mortality rate according to the presence or absence of LC.

The overall mortality of all patients analyzed in the study cohort was 60%. There was no difference in mortality between male and female patients (*p* = 0.212). In the group of patients with LC, 53 died corresponding to a mortality rate of 79.1% (95%-confidence interval [CI]: 67.4%–88.1%). In the group without LC the mortality rate was 44%. The presence of LC was associated with a significant increased risk of mortality (Odds ratio (OR): 4.809) ([95%-confidence interval (CI): 2.32–9.97]; *p* < 0.001) as shown in Figure 1.

As mentioned above, a majority of patients with LC (88%) showed a decreased liver function categorized in the child-pugh class C. In this group only 9 out of 59 patients survived resulting in a mortality rate of 84.7%.

**Figure 2:**
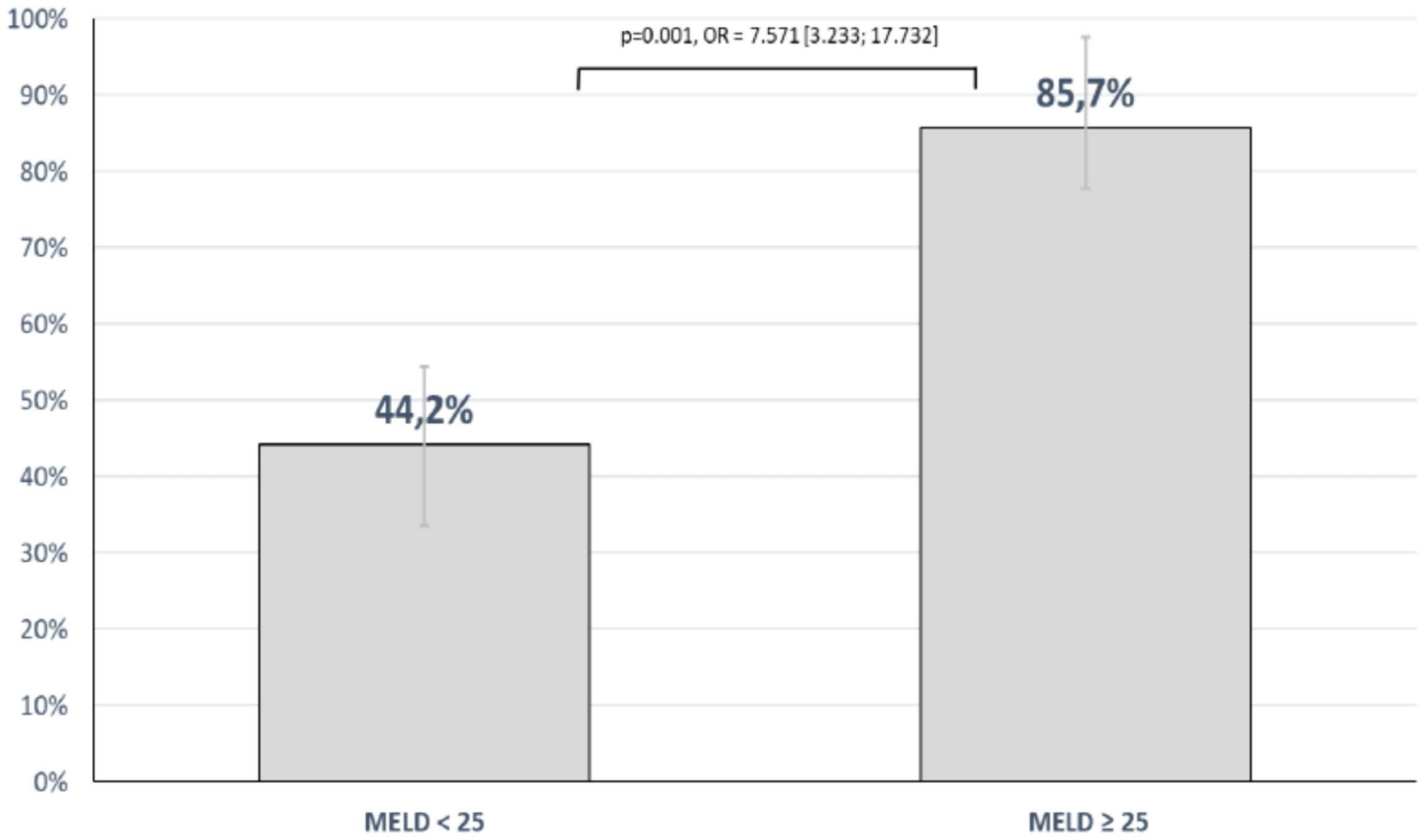
Mortality rate according to the MELD score.

The mean model of end stage liver disease (MELD) score in the patients with LC was 32.3 (±7.8). Patient with LC that survived Pneumocystis jirovecci infection had a mean MELD Score of 23.1 (±7.8), while non-survivors showed a mean MELD score of 34.8 (±5.8) (*p* < 0.001). There was a significant correlation of MELD score and mortality (*r* = 0.612, *p* < 0.001). The mortality rate of patients with a MELD score above 25 was significantly higher than in patients with a MELD score below 25 with an OR of 7.571 (95%-CI: 3.23–17.73; *p* < 0.001) (see Figure 2).

Interestingly, immunosuppression other than LC was not associated with an increased mortality risk (OR: 1.103; 95%-CI: 0.57–2.11; *p* = 0.767) (see Table 2).

### 3.3 Fungal pathogen concentration, and mortality

As demonstrated in Table 2. the majority of patients had a Pneumocystis jirovecci concentration in the analysed specimens between 100 and 10.000 pathogens (63%). A minority of patients (24%) had a fungal load above 10.000 pathogens in the examined specimens. Regarding the outcome of the patients, the fungal pathogen load had no significant influence (*p* = 0.365). Furthermore there was no correlation between the kind of examined specimen and the mortality risk (*p* = 0.218).

### 3.4 Treatment

In this study, there were no therapeutic criteria for Pneumocystis jiroveccitherapy available. Table 1. summarizes the treatment of the patient cohort after diagnosis of Pneumocystis jirovecci infection in correlation with the pathogen concentration. Besides the pathogen concentration, a high sequential organ failure assessment (SOFA) score led to the initiation of a specific therapy (OR of 1,1,522 [95%-CI: 1.05–1.26]; *p* = 0.002).

**Figure 3:**
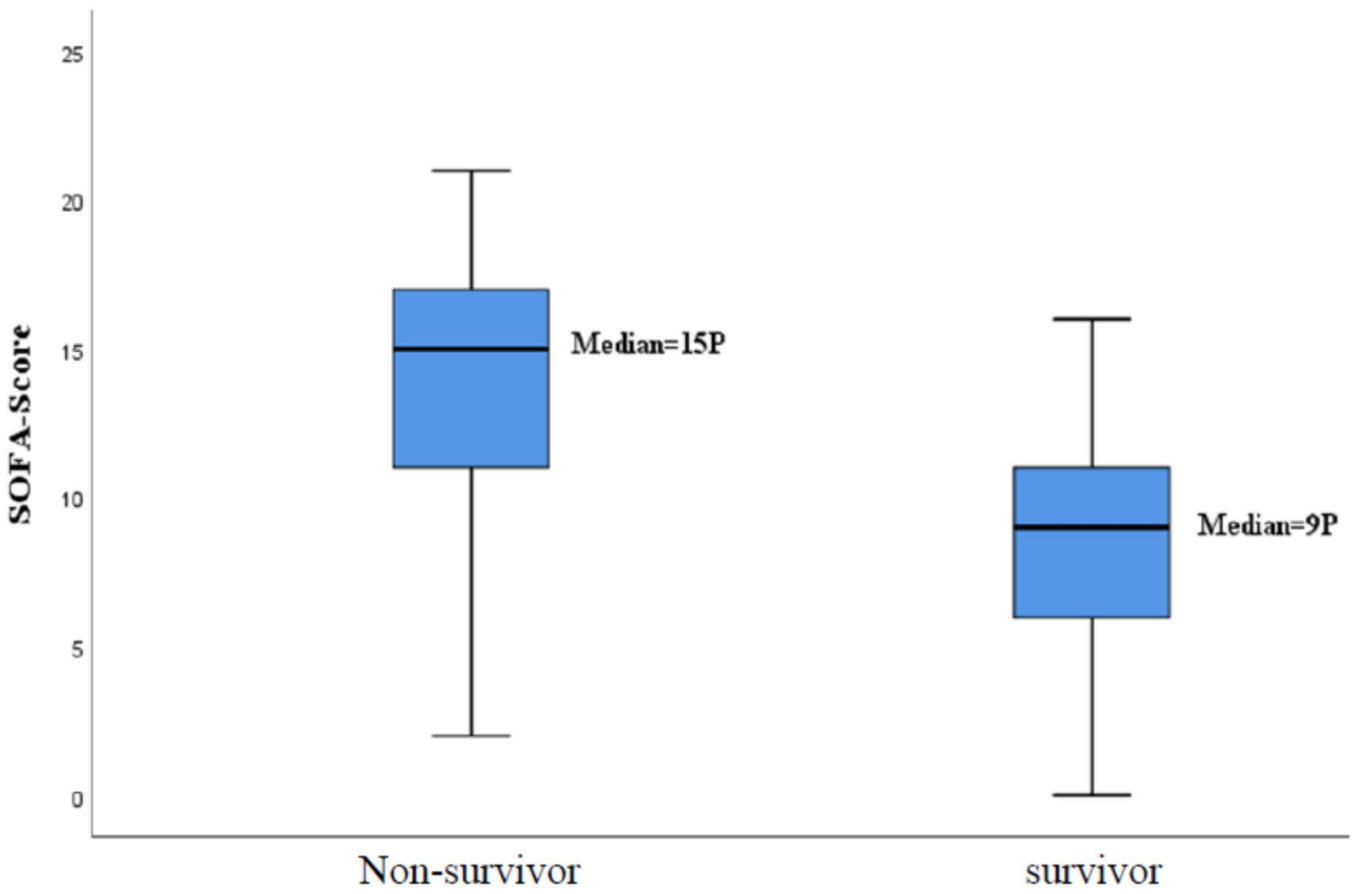
Mortality rate according to the SOFA-score.

In total, 104 patients (70%) received a Pneumocystis jirovecci specific therapy. In the group of the non-survivors (mean: 13.9 ± 4.2) the SOFA score was significantly higher than in the group of the survivors (mean: 8.3 ± 4.1) (*p* < 0.001) as shown in Figure 3. The same accounts for patients with LC. Patients with LC (mean: 15.3 ± 3.3) had a higher SOFA score than patients without this disease (mean: 8.7 ± 4). Despite of the initiated Pneumocystis jirovecci therapy, 104 patients that received a specific therapy 71 (68%) died, while 19 (40%) out 47 with no therapy died (*p* = 0.006). This suggests that patients receiving a therapy had a higher mortality risk (OR = 3.171; 95%-CI: 1.553–6.475). A comparable result was seen for patients with LC. Patients with LC receiving a specific therapy died in 85% of the cases, while 63% of patients with LC and no therapy died (OR = 3.417; 95%- KI: 0.999; 11.682) (see Table 3).

**Table 3:** Association between therapy and outcome in patients with LC.

LC	Patients (*n*)	No therapy	Specific therapy	
Survivor	14 (21%)	7 (37%)	7 (15%)	OR = 3.417 [95%-CI: 0.999–11.682]
Dead	53 (79%)	12 (63%)	41 (85%)	

## 4 Discussion

To our knowledge, this is the largest study analyzing Pneumocystis jirovecci infection in patients with advanced LC. In our study, patients with LC and Pneumocystis jirovecci infection had a significantly higher mortality compared to infected patients without LC. It has to be noted that up to now only a few case series described Pneumocystis jirovecci infection in patients with LC (13, 14). In those case series, it has been suggested that LC might be an independent risk factor for PCP. Our data clearly indicate a heightened susceptibility to fungal infections. In our study, the majority of patients with LC presented with advanced LC with a mean Meld Score of 32.3. Furthermore, our data show a significant correlation of MELD score and mortality (*r* = 0.612, *p* < 0.001). We were also able to show that a cut-off of a Meld Score above 25 can identify patients with the highest mortality risk.

Advanced liver disease itself, as in our cohort can be considered as a cause of acquired immunodeficiency and a higher mortality (7, 15). There are up to now only a few case reports that underline the possible relevance of immunodeficiency in decompensated LC and the risk for opportunistic infections. In case of an acute alcoholic hepatitis, especially under treatment with steroids the threat of PCP is better accepted, although with a low evidence (16). A weakness of the immune system in patients with LC is well accepted and described as CAID (17). But this has been shown mainly for bacterial infections and those infections are the main triggers for an acute on chronic liver failure (ACLF). In our cohort patients with liver cirrhosis had high MELD score, Child-Pugh score and SOFA score values which suggest high grade systemic inflammation in patients with decompensated cirrhosis. Patients with end stage liver disease and sepsis may develop acute-on-chronic liver failure (ACLF), that results in an immune exhaustion, characterized by a dysregulation of immune effector cells (6, 8). This includes a reduced potential of CD4+ lymphocytes and NK-cells in cirrhosis (18, 19). Risk for Pneumocystis jirovecciinfection is associated with low lymphocyte count and mortality of Pneumocystis jirovecci infected patients is associated with both, low lymphocyte and low NK cell counts (20, 21). Therefore, the immune-dysfunctional pattern of patients with sepsis in advanced liver disease may support Pneumocystis jirovecci infection. Even a low colonization with Pneumocystis jirovecci, as in some patients of our study especially in context with invasive ventilation represents a serious risk for PCP. Our results clearly show that advanced LC is a risk factor for Pneumocystis jirovecciin the airways associated with a high mortality. As a clinical and therapeutic consequence, prophylactic therapy as for other immunodeficient patients should be considered at an early stage of the infection.

Our patients were extremely fragile, and the high mortality rate in our study could be related not only to Pneumocystis jirovecci, but to the severe co-morbidities and organ failures. This is underlined by the fact that patients with a high SOFA score independently of the diagnosis LC showed the highest mortality rate. Other reasons might be concomitant infections and/or liver transplant delay. Of note, the mean MELD score of the patients with LC in our study was 32 points, which correspondents with a 90-day mortality of about 60% (22, 23).

Our study has several limitations, mainly due to its retrospective character. Thus, we cannot provide data about the immune status of our patients. Furthermore, there was no specific therapy protocol at which point and for what criteria specific therapy for Pneumocystis jirovecci had to be started. In our study therapeutic decision was based on clinical decisions and correlated with microbiological diagnostics and existing clinical risk scores. Furthermore, in our study patients from two different ICU wards were included. Therefore, the composition of the patient population is heterogeneous with a potential bias for patient outcome.

The strength of our study lies in the substantial number of patients with advanced liver cirrhosis (LC) and Pneumocystis jirovecci infection. Our data clearly demonstrate that Pneumocystis jirovecciinfection is a serious opportunistic infection in patients with LC. Additionally, our findings support the concept of cirrhosis-associated immune dysfunction (CAID) and show the increased susceptibility of patients with liver cirrhosis to fungal infections. This susceptibility was correlated with the Child-Pugh and MELD scores. We established a MELD score cut-off above 25 to identify patients with the highest risk of mortality.

In conclusion, our study demonstrates that Pneumocystis jirovecci infection in patients with advanced liver cirrhosis is associated with high mortality. Therefore, Pneumocystis jirovecciinfection should be included in the differential diagnosis for patients with advanced liver cirrhosis and acute-on-chronic liver failure (ACLF). We recommend initiating early antifungal Pneumocystis jirovecci treatment for this vulnerable patient cohort.

Furthermore, we emphasize the importance of early recognition of immune dysregulation as a consequence of cirrhosis and the early identification of Pneumocystis jirovecci infection. We also underscore the role of early treatment of Pneumocystis jirovecci infections in cirrhosis to improve patient outcomes in both transplant and non-transplant settings.

Our data contribute a new phenotypic manifestation, specifically Pneumocystis jirovecciinfection, to the mechanisms of cirrhosis-associated immunosuppression and may lead to immune-targeted therapies to reduce susceptibility to infection and mortality in patients with cirrhosis.

## Data Availability

The original contributions presented in the study are included in the article/supplementary material, further inquiries can be directed to the corresponding author.

## Supplementary material

SUPPLEMENTARY FIGURE S1 Genesis of liver cirrhosis (MAFLD, metabolic-dysfunction associated fatty liver disease; PBC, primary biliary cirrhosis; SSC, secondary sclerosing cholangitis; AAT, alpha-1 antitrypsin deficiency).
