# Supplementary material for: Liver cirrhosis is a risk-factor for Pneumocystis jirovecii associated mortality
Source: Front Med (Lausanne). 2024 Oct 9;11:1474835. doi: 10.3389/fmed.2024.1474835 (PMC11496259; doi:10.3389/fmed.2024.1474835)
Supplement: SUPPLEMENTARY FIGURE S1 — Genesis of liver cirrhosis (MAFLD, metabolic-dysfunction associated fatty liver disease; PBC, primary biliary cirrhosis; SSC, secondary sclerosing cholangitis; AAT, alpha-1 antitrypsin deficiency). [file Data_Sheet_1.pdf]

Supplementary data:

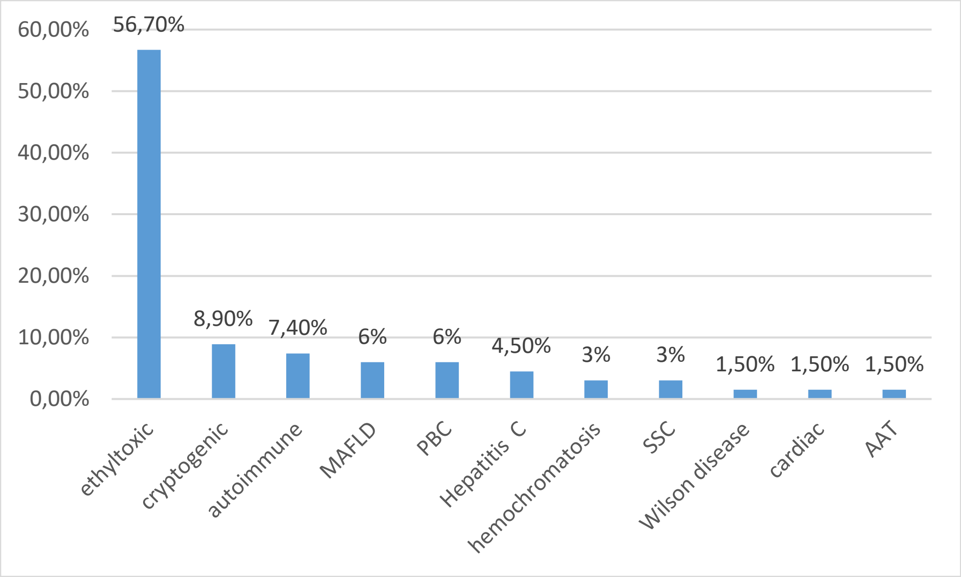

Figure 4.: Genesis of liver cirrhosis (MAFLD: metabolic-dysfunction associated fatty liver disease; PBC: primary biliary cirrhosis; SSC: secondary sclerosing cholangitis; AAT: Alpha-1 antitrypsin deficiency).
